# A Hazard Assessment Method for Waterworks Systems Operating in Self-Government Units

**DOI:** 10.3390/ijerph16050767

**Published:** 2019-03-03

**Authors:** Janusz R. Rak, Barbara Tchórzewska-Cieślak, Katarzyna Pietrucha-Urbanik

**Affiliations:** Department of Water Supply and Sewerage Systems, Faculty of Civil, Environmental Engineering and Architecture, Rzeszow University of Technology, Al. Powstańców Warszawy 6, 35-959 Rzeszów, Poland; rakjan@prz.edu.pl (J.R.R.); cbarbara@prz.edu.pl (B.T.-C.)

**Keywords:** waterworks systems, risk, risk matrix, self-government units

## Abstract

Informing users of waterworks systems about the quality of tap water is an obligatory trend. It should be accompanied by studies on the influence of the risk of threats on public health. Waterworks systems, being included in a critical infrastructure of the city, should be subject to special protection in this respect. In the paper, the authors’ method of assessing threats to people and property from waterworks systems functioning in self-government units (SGUs), is proposed. Four categories of factors affecting the risk of threat to tap water consumers were assumed: the frequency or the probability of exposure—*P*, financial losses—*C*, damages to peoples’ health—*HL*, the degree of the security—*S*. Based on this, a four-parametric risk matrix was developed. It was assumed that risk is a function of the parameters mentioned above: *r* = *f*(*P*, *C*, *HL*, *S*). For every parameter the five-parametric weight scale was assumed. An example of applying the method is presented. The proposed method should be an important element of water safety plans. It can also be adopted for other municipal systems subject to SGU.

## 1. Introduction

Water supply systems belong to the so-called critical infrastructure of the state in accordance with the European guidelines. Therefore, they should be subject to special protection. Council Directive of the European Union 98/83/EC on the quality of water intended for human consumption, with subsequent amendments in 2003, 2009 and 2015, states that a balance should be struck between protection against risks caused by both chemical and microbiological agents and that the parameter values for water intended for human consumption, in the light of their future assessments, should be set on the basis of public health considerations using risk assessment methods [1]. Article 6 states that appropriate measures will be taken to reduce or eliminate the risk of non-compliance with the quality parameters of water, and consumers must be duly informed about any corrective actions related to the reduction or elimination of the above-mentioned risk. Article 8 imposes an obligation to analyse the risk to human health if water does not meet the quality parameters. Currently, work is underway on a new directive which introduces an obligation for entities responsible for supplying water to develop and implement the so-called water safety plans based on analyses and risk assessments [2]. Also the European Union recommend member countries to implement standards regarding drinking water supply security concerning guidelines for risk and crisis management through standards EN 15975-1 and -2 Part 1: Crisis management and Part 2: Risk management [3,4].

The Commission pays particular attention to the following priorities in the water supply management process:

- application of a risk-based approach in the design, construction and operation of water supply systems,
- increasing the transparency of procedures and providing consumers with access to up-to-date information on water quality and potential threats,
- minimizing potential damages.

According to regulations regarding the quality of water intended for human consumption, the competent, district or border sanitary inspectors issue periodic assessments of water quality. The authorities of the State Sanitary Inspectorate perform water quality and health risk assessment in the given area. The assessment includes a list of water producers supplying the population and providing water from individual intakes, as part of economic activity, to public buildings and residential buildings. In addition, information is provided about:

- the volume of water production supplied by individual producers and the method of water treatment,
- the number of people supplied with water,
- water quality, the way it is treated and disinfected, if this is used,
- exceeding the limit values of water quality parameters with an indication of their impact on the health of consumers,
- reported adverse reactions related to water consumption in the given area,
- administrative proceedings in the field of water quality,
- repair activities carried out by water and sewage companies.

Taking the right decisions for water treatment process or warning people of poor water quality always requires a certain time advance. A very important issue related to ensuring the safety of water supply operation includes the measurement of pressure and flow rate in the water supply network, as well as monitoring of water quality in treated water tanks and in selected points of the water supply network [5,6,7]. The sooner the system operator receives information about a threat, the greater the possibility to make the right decisions, as to restore the reliable water supply to recipients [8,9]. It should be noted that proper operation of a water network should include the following steps: conducting inspections of fittings of the water supply network through the maintenance or replacement, as well as current repairs of the water supply network and its renovation, reconstruction and replacement [10,11,12,13,14,15].

The paper presents proposals for a risk analysis and assessment method based on a four-parameter matrix, including the assessment of risks for people and property from water supply systems operating in self-government units (SGU). The method should be applied in analysing risk of lack of water supply in crisis situations, taking into account the specificity of the local collective water supply system (CWSS).

## 2. Material and Methods

### 2.1. Risk Analysis Approach

The risk associated with the operation of collective water supply systems means the possibility of an undesirable event having an impact on the achievement of the objective, which is the safe supply of water for consumption. According to the international ISO standard [15,16] and normative guidelines, the risk assessment consists of its identification, analysis, assessment and evaluation. People and Property Hazard Analysis (PPHA) method that can be used to estimate losses requires determining the upper limits of tolerable and controlled risk [17]. The goal of risk management is to bring its size to the level at least tolerable and, preferably, to the level of As Low As Reasonably Practicable (ALARP—which means “as low as reasonably feasible”) [18]. The novelty of the proposed method is the separation of material and human losses [17].

In crisis management a proper risk assessment is the basis for taking actions to ensure safety efficiently and effectively. Efficient actions should be understood as full achievement of the set goals. Effective means achieving certain results. Risk analysis methods are being developed mainly to meet the needs of safety engineering [19,20,21,22,23,24,25,26,27,28]. This, in turn, implies the use of risk assessments in crisis management. The Act on Crisis Management obliges the assessment of risk in relation to, at least, human losses (fatalities, missing people, injured people requiring hospitalization and qualified medical help) and property losses [29,30]. The classic definition of risk shows that its assessment consists in multiplying the likelihood or frequency of occurrence of a threat by the projected losses [31,32,33]. Risk assessment requires determining the value of both factors. The joint consideration of human damages and material losses raises ethical concerns [34,35]. For this reason, we should categorize the risk associated with material losses and with human damage separately. People and Property Hazard Analysis (PPHA) method assumes the adoption of five-point scales for individual risk factors–very little, little, medium, large, very large) [17,36,37]. The ranges of values in the distinguished categories were derived from literature data from EU countries and expert experience of the authors of the article in the implementation of the WSP in water supply systems. The considered method is a practical implementation in the operation of small water supply systems. The risk analysis of water supply systems in big urban agglomerations should include uncertainty analysis, which must be strictly taken into account [38,39,40]. The infrastructure risk is defined as the product of the probability of system failure and associated costs of returning the system to service [41]. As to perform effective risk assessment and management, the analyst must understand the system and its interactions with its environment [42,43], and this understanding is requisite to modeling the behaviour of the state of the system under varied probabilistic conditions [44].

### 2.2. Probability Estimation Methodology

The estimation of probability of threat can be made based on the modified Bernoulli distribution [17,19]. The classic Bernoulli formula for the probability of obtaining *k* of successes in n samples is calculated by the formula:

(1) $$P\left(k\right)=\left(\frac{n}{k}\right)p^{k}\cdot q^{n-k},$$

where *P* is the probability of success, *q* = 1 − *p* is the probability of failure, *k* is the number of successes, *n* is the number of tries.

Assuming that:

*P*(*A*) = 1 − *P*(*A*_1_), (2)

where *P*(*A*) is the probability of occurrence of a given threat, and *P*(*A*_1_) is the probability of no occurrence of a threat.

In turn:

(3) $$P\left(A_{1}\right)=\left(\frac{n}{k}\right)p^{k}\cdot q^{n-k},$$

and for the event *A*_1_, *n* = *k* and *p* = 1 − *q*, then the formula (2) takes the form [17]:

(4) $$P\left(A\right)=1-\left[\left(\frac{n}{k}\right)p^{k}\cdot q^{n-k}\right]=1-\left[1\cdot {\left(1-q\right)}^{n}\cdot q^{o}\right]=1-{\left(1-q\right)}^{n},$$

where *q* is the frequency of occurrence of threat *A*, it is the value obtained from experience and can be identified with a posteriori probability.

When determining the time perspective for which the probability of threat is calculated, not only the assumed time period of prospective N analysis should be taken into account, but also the time that has elapsed since the last year when threat occurred. Thus [17]:

(5) $$n=N+\left(n_{1}-n_{2}\right),$$

where *n_1_* is the year in which the analysis is carried out, *n_2_* is the year in which the last threat occurred, and *N* is time period of prospective analysis.

Static probability is identified by a statistical estimate having a constant value for a given observation period, e.g., static probability determining the occurrence of risk once every 10 years equals *P’* = 0.1. In contrast, dynamic probability is variable over time and dependent on the initial value, which is the static probability. Table 1 presents the scale of categories of frequency and static and dynamic probability of the occurrence of an undesirable event with the point weights for individual categories [17].

### 2.3. Methodology for Estimating Material Losses

Estimating material losses being a result of an undesirable event is a complex and multifaceted task [17]. The valuation of assets of people, enterprises, real estate, etc. is subject to current conjuncture. In the work as the measure of losses the interest on the SGU income was assumed.

Table 2 presents the scale of the categories of material losses with the point weights for particular categories [17].

### 2.4. Methodology for Estimating Human Losses

The indicator used in analyses and assessments of accidents at work was adopted. The accident frequency rate indicates the number of undesirable events per 1000 people employed in a given sector of the economy. By analogy, the number of undesirable events per 1000 users of the public waterworks, was adopted. Three types of human losses were distinguished [17]:

- providing qualified medical assistance—*HL_pma_*,
- required hospitalization—*HLr_h_*,
- deadly descent—*HL_dd_*.

Table 3 presents the scale of the category of nuisances of human losses with the point weights for particular categories [17]. The loss category is always taken according to the largest value of estimated losses.

The issue of estimating the risk associated with the estimation of the risk of human life threat is very sensitive and difficult in social perception, therefore we have presented, in addition to deadly descents, category of human losses as necessity of required hospitalization and providing qualified medical assistance. We are aware that comparing human and material losses is unethical, but the descriptions of spectacular water supply failures confirm that these types of losses are smaller. We have adopted the interpretation that the occurrence of losses related to death accidents should be considered as an unacceptable risk, independent of the final result obtained, and the transfer of the received risk to the unacceptable risk interval. It should be noted that the risk assessment requires each time performing a risk analysis.

### 2.5. Four-Parameter Risk Matrix

In the method the risk is determined according to the formula [23,24]:

(6) $$r=\frac{P\cdot C\cdot HL}{S},$$

where *P* is the probability of a threat, *C* are material losses, *HL* are human losses, and *S* is security category. Table 4 presents a four-parameter risk matrix, which, based on formula (6), allows one to estimate the amount of risk.

Weights for parameters P, C and F are taken according to the Table 1, Table 2 and Table 3. For the parameter S, depending on the degree of water supply security, the following questionnaire can be used to estimate the category and point weight [23,24]. The proposed survey to pre-estimate the level of security of the CWSS [23,24]:

- How often is the raw water quality monitored?
  - every day—1 point,
  - periodically (once a month, once a quarter)—5 points,
  - randomly, if a threat is found—10 points,
- How often is the treated water monitored?
  - every day—1 point,
  - periodically (once a week, once a month)—5 points,
  - randomly, if a threat is found—10 points,
- Does CWSS have a protection and warning station if it takes surface water?
  - yes—1 point,
  - no—3 points,
- Are the design requirements for the water intake protection zones implemented?
  - in total—1 point,
  - with some exceptions—3 points,
  - there are difficulties, e.g., economic, legal, etc.—6 points,
- Is it possible to provide alternative water supply (emergency wells, two or more water supply sources)?
  - yes—1 point,
  - partial—4 points,
  - no—10 points,
- Does the water supply company:
  - have own specialized service for removing network failures—1 point,
  - have a contract with an economic entity that intervenes if necessary— 3 points,
  - search for a contractor to remove failures— 10 points,
- The emergency volume of treated water in water tanks is:
  - 0 ÷ 10% *Q_dmax_*—6 points,
  - 10 ÷ 50% *Q_dmax_*—3 points,
  - over 50% *Q_maxd_*—1 point,
- If the sum of points from the questionnaire is:
  - 7 ÷ 10—high level of security—*w* = 3,
  - 12 ÷ 34—medium security level—*w* = 2,
  - above 34—low level of security—*w* = 1,

In the proposed method, due to the objectives of the SGU, taking into account the formula (6), three risk levels are distinguished. Determined levels of risk were result of a decision-making process, as to prioritize what action should be performed.

- tolerated risk—from 0.33 to 5,
- controlled risk—from 5.33 to 15,
- unacceptable risk—from 16 to 125.

The risk value criteria were assumed on the basis of literature data from EU countries and the authors’ experience in the implementation of the WSP in water supply systems. The three point scale is widely used in different technical systems. In the case of uncertain data fuzzy set theory may be used, which is presented in the work [38,39]. The obtained unacceptable risk means, that an immediate action should be taken to reduce this value of risk category. In case of tolerable risk no extra actions are required. Controlled risk means the intermediate level, which means that the system is permitted to function but under the condition that modernization or renovation will start, according to concept of As Low As Reasonably Practicable (ALARP) proposed by Health and Safety Executive.

## 3. Application Examples

### 3.1. 1st Application Example

In 2015, the probability of occurrence of a threat of contamination of the source of surface water intake in the water intake, was estimated. During 30 years (since 1985), contamination occurred 5 times and the last time in 2014. What is the probability of contamination from a perspective of 5 years?

(7) n=5+(2015−2014)=6,

(8) $$q=\frac{5}{30}=0.1667,$$

(9) $$P\left(A\right)=1-{\left(1-q\right)}^{n}=1-{\left(1-0.1667\right)}^{6}=1-0.3341=0.6659,$$

The probability of water contamination in 2017 was re-estimated, also with the perspective of the next 5 years. The last threat occurred unchanged, in 2014:

(10) n=5+(2017−2014)=9,

(11) $$q=\frac{5}{32}=0.15625,$$

(12) $$P\left(A\right)=1-{\left(1-0.15625\right)}^{9}=1-0.2167=0.7833,$$

The probability of water contamination in 2015–2020 was 0.6659 and in 2017–2022 it increases to 0.7833. According to Table 1, this is category large with weight *w* = 4 (dynamic probability).

### 3.2. 2nd Application Example

As a result of the failure of the water supply network and the lack of power supply in the water pumping station, the average annual losses were estimated on the level of 0.45% of the SGU annual expenditures. For such a value, according to Table 2, the value of estimated material losses is in the category very little with the weight w = 1.

### 3.3. 3rd Application Example

The commune uses a group waterworks LM = 5000 people. For an undesirable event related to the secondary water pollution in the water supply network, it was estimated F_pl_ = 50, F_h_ = 5 and F_zs_ = 0.

- the number of people who should be given qualified medical help is:
  (13) $$\frac{LM\cdot F_{pl}}{1000}=\frac{5000\cdot 50}{1000}=250\;people,$$
- the number of people hospitalized is:
  (14) $$\frac{LM\cdot F_{h}}{1000}=\frac{5000\cdot 5}{1000}=25\;people,$$

For such values according to Table 3, the value of estimated human losses corresponds to category large with the weight *w* = 4.

### 3.4. 4th Application Example

Using the data contained in examples 1-3 weights for the individual risk parameters are:

- *P* − *w* = 4,
- *C* − *w* = 1,
- *HL* − *w* = 4,
- *S* was estimated on the basis of the questionnaire for the security parameter, the sum of 21 points means *w* = 2.

Based on Equation (6), the estimated risk is r = 8. The analysed risk related to the risk of contamination of tap water within the analysed SGU is at the controlled level.

## 4. Discussion of Results

Currently, a water supply company is obliged to inform about the deterioration of water quality a competent state sanitary inspector and a community head, mayor or city president, within no more than seven working days. The scope of information included in the application for consent to the derogation was expanded in the field of:

- reasons why water of the required quality cannot be delivered,
- justification along with an indication of actions aiming to ensure the right quality water,
- a study analysis prepared by a scientific institution dealing with public health, regarding the impact of the derogation (concentration and duration) on the health of tap water consumers.

In addition, an obligation to provide a systematic (every 3 months) detailed report on corrective actions taken and actions planned to be taken in the next reporting period, was introduced. Now, information for consumers about water quality also includes data on granted consents to deviation from the acceptable parameters:

Standard information for residents about water quality should include:

- the area of the commune covered by water research,
- the area of the commune not covered by research with an indication of the reasons,
- threats resulting from the lack of water quality tests,
- defining activities that should be taken to protect health against contaminated water.

According to the authors, the method can be used in SGUs of up to 50,000 residents. For the probability of occurrence of an undesirable event: poor water quality or lack of supply (P), material losses (C), population health losses (HL), 5-stage scales were used and for the degree of security of the water system (S) a 3-stage scale was used. The adoption of a 3-stage scale in relation to security (S) results from the fact that for small and medium-sized CWSS such systems are not very expanded. To characterize the degree of security (S) we use the questionnaire with 7 questions.

## 5. Conclusions

- Water supply systems belonging to the critical infrastructure require a detailed risk analysis in view of the possibility of a crisis situation, taking into account rapid response plans to ensure the delivery of water to consumers.
- The method of risk analysis and assessment presented in the work can be used as part of the risk assessment for the needs of water safety plans. Its advantage is the possibility of adapting to the specifics of local CWSS. The method presents a detailed way of assessing the possibility of threats as well as a method of loss assessment.
- People and Property Hazard Analysis (PPHA) method is a kind of development of the Preliminary Hazard Analysis (PHA) method. It allows for the inclusion of human and material losses separately in the adopted planning perspective by the category of determining the probability of an undesirable event occurrence.
- The method also takes into account the degree of security of the water supply system against threats, including aspects of the multibarrier system. In this aspect the use of the authors’ questionnaire to estimate the security parameter is proposed.
- The method of analysis and risk assessment proposed in the work is based on the authors’ four-parameter risk matrix.

## Figures and Tables

**Table 1:** Categories of frequency and probability of threat occurrence.

Category/Point Weight—*w*	Static Frequency—*f*	Static Probability—*P’*	Dynamic Probability—*P*
very little/*w* = 1	from once every 100 years up to once every 50 years	0.01–0.02	<0.1
little/*w* = 2	from once every 50 years up to once every 20 years	0.02–0.05	0.1 ≤ *P* < 0.4
medium/*w* = 3	from once every 20 years up to once every 5 years	0.05–0.2	0.4 ≤ *P* < 0.7
large/*w* = 4	from once every 5 years up to once every 2 years	0.2–0.5	0.7 ≤ *P* < 0.9
very large/*w* = 5	from once every 2 years to at least once a year	> 0.5	*P* ≥ 0.9

**Table 2:** Category of material losses.

Category/Point Weight—*w*	The Amount of Material Losses—*C*
Very little/*w* = 1	up to 0.5% of annual budget expenditure, which is the minimum value of the specific reserve designated for the implementation of own tasks in the field of crisis management PPHA
Little/*w* = 2	up to 5% of annual PPHA expenses
Medium/*w* = 3	up to 15% of annual PPHA expenses
Large/*w* = 4	over 15% of annual PPHA expenses
Very large/*w* = 5	no possibility of adopting a budget for the next year due to exceeding the individual PPHA debt ratio

**Table 3:** Category of human losses.

Category/Point Weight—w	Human Loss Rate		
Very/little/*w* = 1	*HL_pma_* ≤ 5	*HL_rh_* = 0	*HL_dd_* = 0
Little/*w* = 2	*HL_pma_* ≤ 25	*HL_rh_* ≤ 2	*HL_dd_* = 0
Medium/*w* = 3	*HL_pma_* ≤ 100	*HL_rh_* ≤ 20	*HL_dd_* ≤ 0.05
Large/*w* = 4	*HL_pma_* ≤ 250	*HL_rh_* ≤ 100	*HL_dd_* ≤ 0.5 *
Very large/*w* = 5	*HL_pma_* > 250	*HL_rh_* > 100	*HL_dd_* > 0.5 *

* Notes: the occurrence of losses related to death accidents should be considered as an unacceptable risk, independent of the final result obtained, and the transfer of the received risk to the unacceptable risk interval.

**Table 4:** The four-parameter matrix.

***HL***	***P***														
	**1**														
	***C***														
	**1**			**2**			**3**			**4**			**5**		
	***S***														
	**3**	**2**	**1**	**3**	**2**	**1**	**3**	**2**	**1**	**3**	**2**	**1**	**3**	**2**	**1**
**1**	0.33	0.50	1.00	0.67	1.00	2.00	1.00	1.50	3.00	1.33	2.00	4.00	1.67	2.50	5.00
**2**	0.67	1.00	2.00	1.33	2.00	4.00	2.00	3.00	6.00	2.67	4.00	8.00	3.33	5.00	10.0
**3**	1.00	1.50	3.00	2.00	3.00	6.00	3.00	4.50	9.00	4.00	6.00	12.0	5.00	7.50	15.0
**4**	1.33	2.00	4.00	2.67	4.00	8.00	4.00	6.00	12.0	5.33	8.00	16.0	6.67	10.0	20.0
**5**	1.67	2.50	5.00	3.33	5.00	10.0	5.00	7.50	15.0	6.67	10.0	20.0	8.33	12.5	25.0
***HL***	***P***														
	**2**														
	***C***														
	**1**			**2**			**3**			**4**			**5**		
	***S***														
	**3**	**2**	**1**	**3**	**2**	**1**	**3**	**2**	**1**	**3**	**2**	**1**	**3**	**2**	**1**
**1**	0.67	1.00	2.00	1.33	2.00	4.00	2.00	3.00	6.00	2.67	4.00	8.00	3.33	5.00	10.0
**2**	1.33	2.00	4.00	2.67	4.00	8.00	4.00	6.00	12.0	5.33	8.00	16.0	6.67	10.0	20.0
**3**	2.00	3.00	6.00	4.00	6.00	12.0	6.00	9.00	18.0	8.00	12.0	24.0	10.0	15.0	30.0
**4**	2.67	4.00	8.00	5.33	8.00	16.0	8.00	12.0	24.0	10.6	16.0	32.0	13.3	20.0	40.0
**5**	3.33	5.00	10.0	6.67	10.0	20.0	10.0	15.0	30.0	13.3	20.0	40.0	16.6	25.0	50.0
***HL***	***P***														
	**3**														
	***C***														
	**1**			**2**			**3**			**4**			**5**		
	***S***														
	**3**	**2**	**1**	**3**	**2**	**1**	**3**	**2**	**1**	**3**	**2**	**1**	**3**	**2**	**1**
**1**	1.00	1.50	3.00	2.00	3.00	6.00	3.00	4.50	9.00	4.00	6.00	12.0	5.00	7.50	15.0
**2**	2.00	3.00	6.00	4.00	6.00	12.0	6.00	9.00	18.0	8.00	12.0	24.0	10.0	15.0	30.0
**3**	3.00	4.50	9.00	6.00	9.00	18.0	9.00	13.5	27.0	12.0	18.0	36.0	15.0	22.5	45.0
**4**	4.00	6.00	12.0	8.00	12.0	24.0	12.0	18.0	36.0	16.0	24.0	48.0	20.0	30.0	60.0
**5**	5.00	7.50	15.0	10.0	15.0	30.0	15.0	22.5	45.0	20.0	30.0	60.0	25.0	37.5	75.0
***HL***	***P***														
	**4**														
	***C***														
	**1**			**2**			**3**			**4**			**5**		
	***S***														
	**3**	**2**	**1**	**3**	**2**	**1**	**3**	**2**	**1**	**3**	**2**	**1**	**3**	**2**	**1**
**1**	1.33	2.00	4.00	2.67	4.00	8.00	4.00	6.00	12.0	5.33	8.00	16.0	6.67	10.0	20.0
**2**	2.67	4.00	8.00	5.33	8.00	16.0	8.00	12.0	24.0	10.6	16.0	32.0	13.3	20.0	40.0
**3**	4.00	6.00	12.0	8.00	12.0	24.0	12.0	18.0	36.0	16.0	24.0	48.0	20.0	30.0	60.0
**4**	5.33	8.00	16.0	10.6	16.0	32.0	16.0	24.0	48.0	21.3	32.0	64.0	26.6	40.0	80.0
**5**	6.67	10.0	20.0	13.3	20.0	40.0	20.0	30.0	60.0	26.6	40.0	80.0	33.3	50.0	100
***HL***	***P***														
	**5**														
	***C***														
	**1**			**2**			**3**			**4**			**5**		
	***S***														
	**3**	**2**	**1**	**3**	**2**	**1**	**3**	**2**	**1**	**3**	**2**	**1**	**3**	**2**	**1**
**1**	1.67	2.50	5.00	3.33	5.00	10.00	5.00	7.50	15.0	6.67	10.0	20.0	8.33	12.5	25.0
**2**	3.33	5.00	10.0	6.67	10.0	20.0	10.0	15.0	30.0	13.3	20.0	40.0	16.6	25.0	50.0
**3**	5.00	7.50	15.0	10.0	15.0	30.0	15.0	22.5	45.0	20.0	30.0	60.0	25.0	37.5	75.0
**4**	6.67	10.0	20.0	13.3	20.0	40.0	20.0	30.0	60.0	26.6	40.0	80.0	33.3	50.0	100
**5**	8.33	12.5	25.0	16.6	25.0	50.0	25.0	37.5	75.0	33.3	50.0	100.	41.6	62.5	125

Notes: 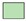 tolerated risk, 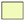 controlled risk, 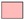 unacceptable risk. Ex. Obtained value r = 0.33, was calculated on the base of categories and values: *P* = 1 (frequency up to once every 50 years), C = 1 (material losses up to up to 0.5% of annual budget expenditure), HL = 1 (providing qualified medical assistance HLpma ≤ 5, no required hospitalization and none deadly descent), and S = 3 (high level of security estimated on the basis of the questionnaire).
